# The long noncoding RNA HOTAIR promotes Parkinson’s disease by upregulating LRRK2 expression

**DOI:** 10.18632/oncotarget.15511

**Published:** 2017-02-19

**Authors:** Sheng Wang, Xuan Zhang, Yuanyuan Guo, Han Rong, Tiebang Liu

**Affiliations:** ^1^ Shenzhen Key Laboratory for Psychological Healthcare, Shenzhen Institute of Mental Health, Shenzhen Kangning Hospital, Shenzhen Mental Health Center, Shenzhen, P. R. China

**Keywords:** Parkinson' s disease, long non-coding RNA, HOTAIR, MPTP, LRRK2

## Abstract

Long noncoding RNAs (lncRNAs) have emerged recently as a new class of genes that regulate cellular processes. HOTAIR (Hox transcript antisense intergenic RNA), an approximately 2.2 kb long noncoding RNA transcribed from the HOXC locus, is upregulated in various diseases. However, the role of HOTAIR in Parkinson's disease (PD) remains unclear. A mouse model of PD was developed by intraperitoneal injection of MPTP. The expression of HOTAIR and LRRK2 were detected in the PD mice and in human neuroblastoma cell lines SH-SY5Y pretreated with MPP+. The effect of HOTAIR on the expression of LRRK2 was examined in SH-SY5Y cells through overexpressing or knockdown of HOTAIR. MTT and flow cytometry assay were performed to measure the cell viability and apoptosis of SH-SY5Y cells. We found that HOTAIR was up-regulated in midbrain tissue of MTPT induced PD mice and in SH-SY5Y cells exposed to MPP+. With the presence of HOTAIR overexpression in SH-SY5Y cells, the expression of LRRK2 was increased compared with that in the control. HOTAIR knockdown showed a protective effect on the cell viability of SH-SY5Y cells pretreated with MPP+. HOTAIR knockdown provided protection against MPP+-induced DA neuronal apoptosis by repressing caspase 3 activity. The finding that HOTAIR promoted PD induced by MPTP could add our understanding of the molecular mechanisms in PD. These findings suggested that inhibition of HOTAIR levels is an effective disease-modifying strategy in PD.

## INTRODUCTION

Parkinson's disease (PD), a neurologic disorder featured by resting tremor, slowness of movements, dementia, gait disturbance and postural instability, holds the second largest patient group among the neurodegenerative diseases [1]. The pathophysiologic mechanisms underlying neurodegeneration are related to mitochondrial dysfunction and oxidative stress, which could cause apoptosis of DA neurons [2–3]. Moreover, several kinds of enzyme, such as leucine-rich repeat kinase 2 (LRRK-2) and parkin (PRKN/PARK2) are thought to be involved in the initiation/development of PD and oncogene DJ-1 may also work in the disease progression.. Multiple agents have been under investigation in clinical trials for PD management, yet, most of them were not promissing [4–5]. Therefore, understanding the molecular mechanisms in the pathogenesis of PD and raising effective therapy targets for treatment options are in serious demand.

LncRNAs are RNA transcripts in length of 200 nucleotides (nt) to ∼100 kilobases (kb) yet not functioning as protein synthesis template [6]. They holds cis- or trans-regulatory capabilities, and more than 1000 lncRNAs are highly conserved in mammalian genome [7–8]. Several well-characterized human lncRNAs have been pointed out to be correlated with multiple biological processes, from epigenetic regulation to immune surveillance, as well as embryonic stem cell pluripotency, and most commonly serving as precursors to small RNAs and regulators of mRNA decay [9–12]. Moreover, recent reports have implicated lncRNA deregulation could be one of the common features of various human diseases including AD, and deregulated lncRNAs therefore may be utilized for AD diagnosis, prognosis or even work out to be potential therapeutic targets [13–15]. Recently, increasing evidence has shown that HOTAIR (Hox transcript antisense intergenic RNA), an approximately 2.2 kb lncRNA transcribed from the HOXC locus, is upregulated in various diseases [16–17]. However, the overall biologic function and the signal pathways of HOTAIR in PD still under identification.

## RESULTS

### The expression of HOTAIR and LRRK2 in the brain of PD mice induced by MPTP

**Figure 1:**
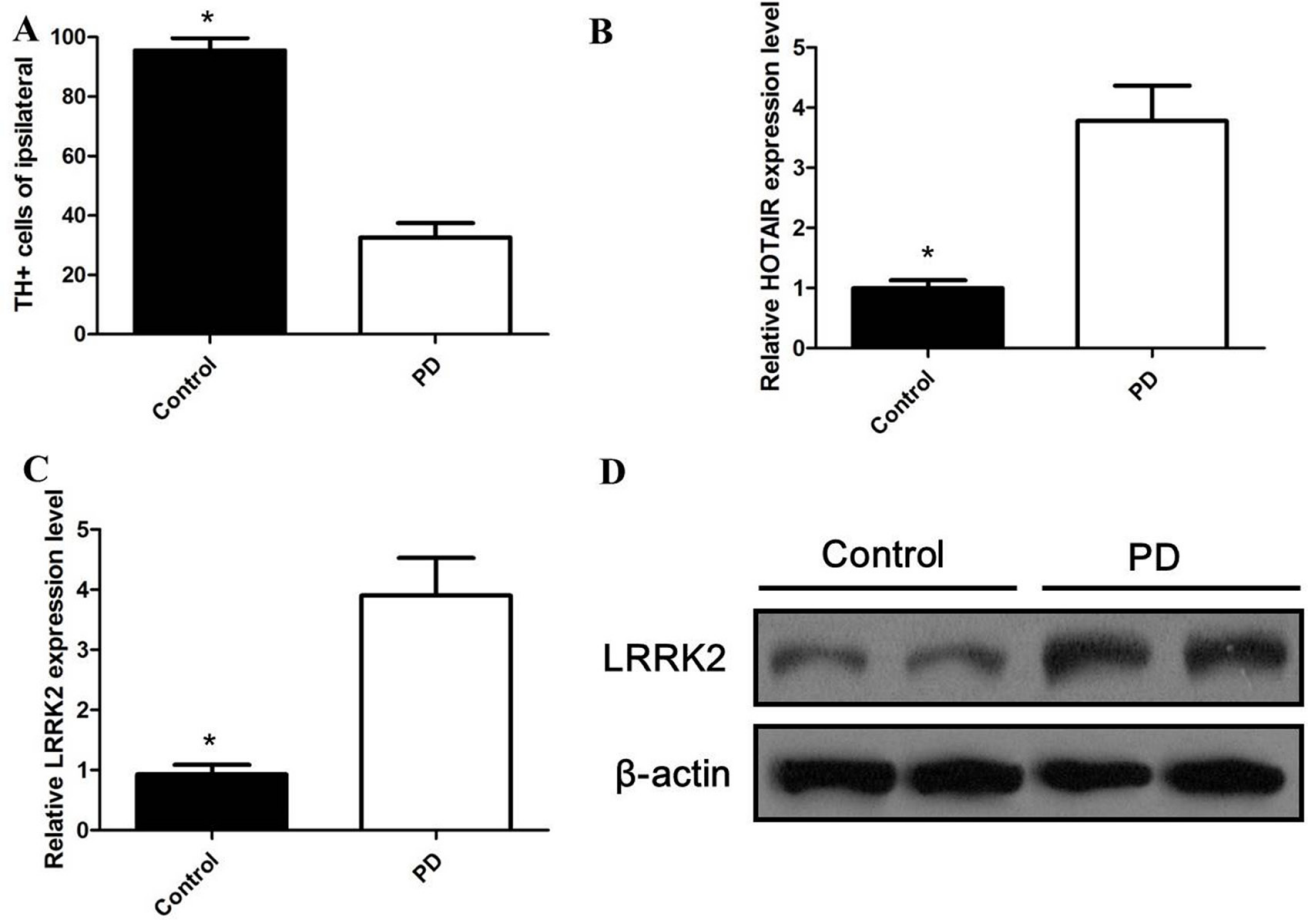
**(A)** TH+ cells of ipsilateral was significantly decreased in PD mice; **(B)** HOTAIR expression in mRNA levels was up-regulated in midbrain of PD mice; **(C)** the mRNA level of LRRK2 was up-regulated in PD mice; **(D)** the protein level of LRRK2 was up-regulated in PD mice.

To detect the expression of HOTAIR and LRRK2 in the brain of PD mice, a mouse model of PD was established through intraperitoneal injection of MPTP. In the PD mice, we observed that the TH+ cells of ipsilateral was significantly decreased in PD mice, compared with that in the control (Figure 1A). The result of qRT-PCR demonstrated that HOTAIR mRNA expression was up-regulated in midbrain of PD mice (Figure 1B). Meanwhile, the mRNA and protein level of LRRK2 were up-regulated in PD mice compared with the controlled group (Figure 1C and 1D).

### The expression of HOTAIR and LRRK2 in MPP+− treated SH-SY5Y cells

**Figure 2:**
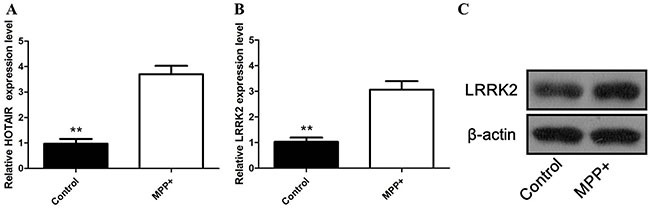
**(A)** the mRNA level of HOTAIR was significantly increased in SH-SY5Y cells pretreated with MPP+; **(B)** the mRNA level of LRRK2 was significantly increased in SH-SY5Y cells pretreated with MPP+; **(C)** the protein level of LRRK2 was significantly increased in SH-SY5Y cells pretreated with MPP+.

To confirm the dysregulation of HOTAIR and LRRK2 in PD mice, the levels of HOTAIR and LRRK2 expression in SH-SY5Y cells exposed to MPP+ was then investigated. As shown in Figure 2A, the mRNA level of HOTAIR was significantly elevated in SH-SY5Y cells pretreated with MPP+. The qRT-PCR and western blot data showed that MPP+ also induced the LRRK2 expression levels in cultured cells (Figure 2B and 2C), indicating that HOTAIR and LRRK2 might be correlated with the development of PD.

### The effect of HOTAIR overexpression on the expression of LRRK2 in SH-SY5Y Cells

**Figure 3:**
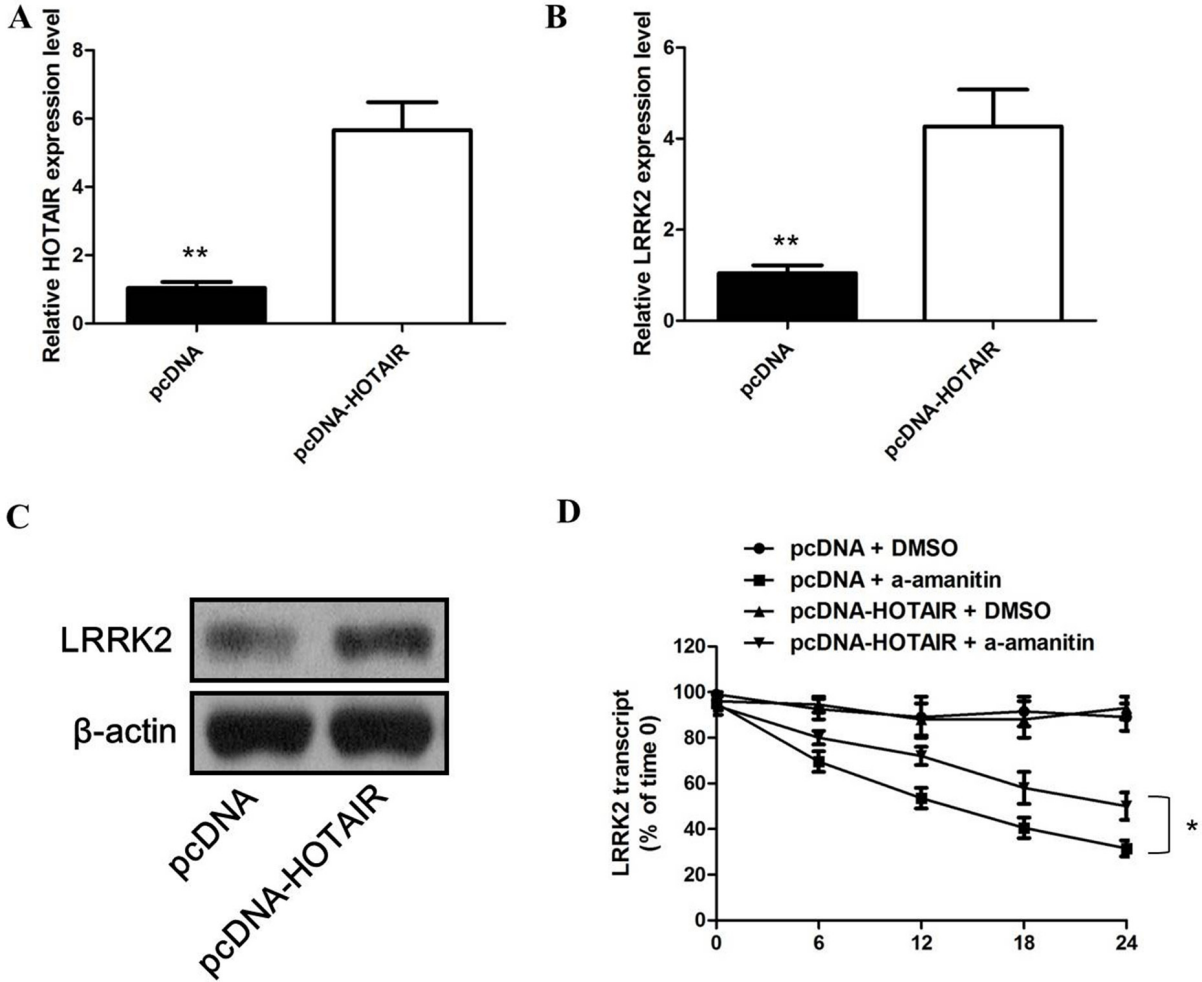
**(A)** the expression of HOTAIR was markedly up-regulated in SH-SY5Y cells transfected with pcDNA-HOTAIR; **(B)** HOTAIR overexpression increased the mRNA level of LRRK2 in SH-SY5Y cells; **(C)** HOTAIR overexpression increased the protein level of LRRK2 in SH-SY5Y cells; **(D)** HOTAIR overexpression significantly inhibited the degradation of LRRK2 mRNA induced by a-amanitin.

SH-SY5Y cells were transfected with pcDNA-HOTAIR to overexpress HOTAIR. As shown in Figure 3A, the expression of HOTAIR was markedly up-regulated in SH-SY5Y cells transfected with pcDNA-HOTAIR than that in the control. At the same time, HOTAIR overexpression increased the mRNA and protein level of LRRK2 in SH-SY5Y cells (Figure 3B and 3C). To test whether HOTAIR regulated the synthesis of LRRK2 mRNA, SH-SY5Y cells were treated with pcDNA + DMSO, or pcDNA-HOTAIR + DMSO, or pcDNA + a-amanitin, or pcDNA-HOTAIR + a-amanitin. The a-amanitin was known to inhibit the synthesis of new mRNA. Here, we found that a-amanitin indeed suppressed the synthesis of new LRRK2 mRNA. While HOTAIR overexpression significantly inhibited the degradation of LRRK2 mRNA induced by a-amanitin (Figure 3D). These data indicated that HOTAIR specifically increased the stability of LRRK2 mRNA and upregulated its expression.

### The effect of HOTAIR inhibition on the expression of LRRK2 and cell viability of SH-SY5Y cells

**Figure 4:**
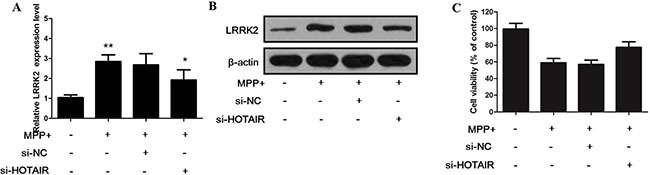
**(A)** HOTAIR knockdown reversed the effect of MPP+ on the mRNA level of LRRK2 in SH-SY5Y cells; **(B)** HOTAIR knockdown reversed the effect of MPP+ on the protein level of LRRK2 in SH-SY5Y cells; **(C)** knockdown of HOTAIR increased the cell viability of SH-SY5Y cells.

To investigate the effect of HOTAIR knockdown on the expression of LRRK2 and cell viability of SH-SY5Y cells, SH-SY5Y cells were divided into four groups: control, treated with MPP+, treated with MPP+ and si-NC, treated with MPP+ and si-HOTAIR. As shown in Figure 4, MPP+ significantly upregulated the expression of LRRK2, and decreased the cell viability of SH-SY5Y cells. However, the interference of HOTAIR expression by si-HOTAIR reversed the effect of MPP+ on the mRNA and protein level of LRRK2 in SH-SY5Y cells (Figure 4A and 4B). Moreover, knocking down of HOTAIR enhanced the cell viability of SH-SY5Y cells (Figure 4C).

### Inhibition of HOTAIR reduces MPP+-enhanced neuronal apoptosis

**Figure 5:**
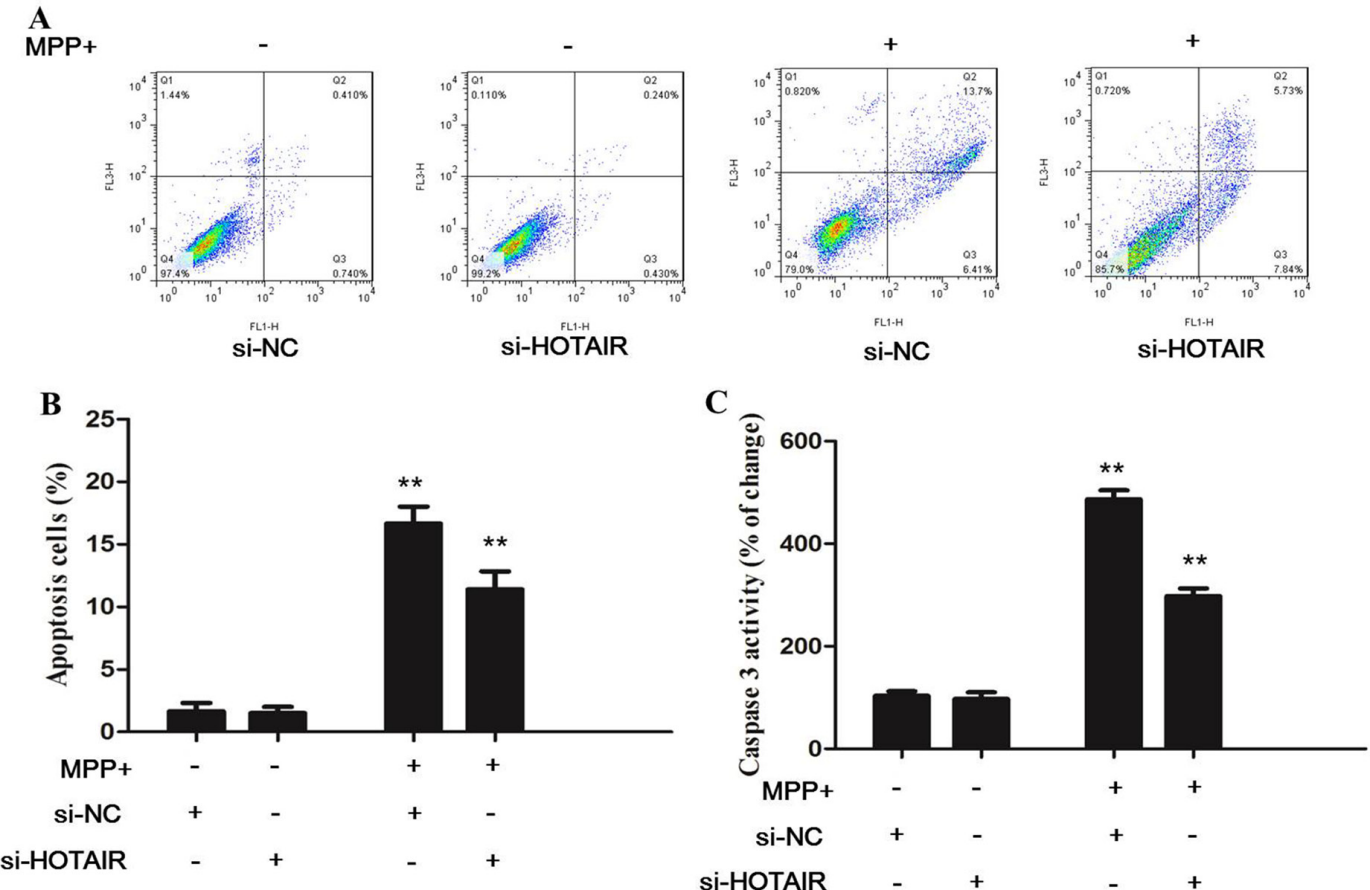
(**A** and **B**) Flow cytometry assay showed that HOTAIR knockdown attenuated the apoptosis of the SH-SY5Y cells induced by MPP+ treatment; **(C)** HOTAIR knockdown inhibits the MPP+−induced SH-SY5Y cell apoptosis by reducing caspase 3 activity.

We further investigated whether HOTAIR regulates neuron survival by orchestrating apoptosis. Flow cytometry assay showed that MPP+ treatment promotes apoptosis of the SH-SY5Y cells, which is attenuated by HOTAIR knockdown (Figure 5A and 5B). In addition, HOTAIR knockdown inhibits the MPP+ induced SH-SY5Y cell apoptosis by reducing caspase 3 activity (Figure 5C).

## DISCUSSION

Previous studies have reported that a large number of ncRNA species take responsibility for the regulation of gene expression in the crucial biologic functions such as neural development, plasticity, and aging [18–19]. Moreover, mutation in LRRK2 has been widely acknowledged as the most common cause of dominantly inherited PD, as well as being one of the risk factors for sporadic PD. Recently, piles of studies suggested that LRRK2 may be participated in membrane trafficking and cytoskeletal dynamic activities, but its exact function has not been clearly sketched out. The proposed theory for LPRK deterioration in the PD pathogenesis is via a gain-of-function mechanism. [20–22].

Several ncRNAs are implicated in PD. Particularly, long noncoding RNAs (lncRNAs) are excessively transcribed and highly expressed in the brain [23]. Newly discoverd evidence suggests lncRNAs hold a critical position in neurodegenerative diseases, specifically in PD [24]. HOTAIR is a member of the human HOX loci-associated 231 ncRNAs and it is apparent that nuclear HOTAIR, by targeting polycomb repressive complex 2, can change the methylation status of H3K27 and manipulate gene expression patterns throughout the genome [25]. A current research reported a scaffold position of HOTAIR serving as an inducer of ubiquitin-mediated proteolysis in the cytoplasm [26]. However, the underlying mechanism it utilized to take part in PD progression remains unclear. In this study, using PD mice constructed by MPTP and SH-SY5Y cell lines treated with MPP+, we observed the up-regulation of HOTAIR in the midbrain of PD mice as well as in SH-SY5Y cells. Under the circumstances of triggering HOTAIR overexpression in SH-SY5Y cells, the expression level of LRRK2 was elevated compared with the control. On the contrary, knocking down HOTAIR demonstrated a protective effect on the cell viability of SH-SY5Y cells treated with MPP+. MPP+ treatment promotes apoptosis of the SH-SY5Y cells, which is attenuated by HOTAIR knockdown.

Neurotoxin MPTP is one of the most common drugs to establish animal models of PD [27]. MPP+ is the active metabolite of MPTP [28]. Neuroblastoma cell line SH-SY5Y is subjected to MPP+ as an *in vitro* model of PD. The pathology and physiology of PD and pharmacology, pharmacokinetic, and drug metabolism have been evaluated using MPTP/MPP+ models. The loss and degeneration of TH+ positive cells is the hallmark feature of PD [29]. In this study, we established a mouse model of PD induced by MPTP and found the considerable reduce of TH+ cells in PD mice. In addition, we found that HOTAIR was up-regulated in midbrain tissue of MTPT induced PD mice and in SH-SY5Y cells exposed to MPP+, suggesting a potential role in the pathogenesis of PD. Mounting evidence demonstrated that the suppression of LRRK2 kinase activity was a potential therapeutic mode for the treatment of neurodegeneration in PD. In this study, we found that HOTAIR specifically increased the stability of LRRK2 mRNA and up-regulated its expression.

The molecular pathogenesis of PD is speculated to be associated with mitochondrial dysfunction and activation of apoptotic cascade. MPP+-induced neuronal death is mediated by the loss of mitochondrial membrane potential. MPP+ treatment promotes apoptosis of the SH-SY5Y cells, which is attenuated by HOTAIR knockdown. Caspase 3 is known as a participating cell-death protease in the execution phase of apoptosis. This study found that HOTAIR knockdown reduces the caspase 3 activity.

In summary, the results presented herein collectively showed that high expression of HOTAIR promoted the onset of PD in the mice model induced by MPTP. In addition, HOTAIR knockdown provided protection against MPP+-induced DA neuronal apoptosis by repressing caspase 3 activity. These findings suggested that inhibition of HOTAIR levels is an effective disease-modifying strategy in PD.

## MATERIALS AND METHODS

### Animals and treatment

Male C57BL/6 mice aged 8–10 weeks were obtained from Chinese Academy of Medical Sciences Laboratory Animal Center (Beijing, China). The animals were maintained on a 12-h light/dark cycle at 25 ± 2°C and 60–70% relative humidity with food and water available *ad libitum*. PD mouse model was established with the administration of neurotoxin 1-methyl-4- phenyl-1,2,3,6-tetrahydropyridine-hydrogen chloride (MPTP-HCl, 20 mg/kg, Sigma–Aldrich, St-Louis, MO, USA) by intra- peritoneal (i.p.) injection for a total of four doses over an 8 h period. PD mice manifests reeling gait, less activity and slow motion. Mice in control group received an equivalent volume of sterile saline solution (0.9%). Mice were killed at selected time points, and their midbrains were used for biochemical analyses. All surgical procedures were performed under anesthesia.

### Cell cultures

Human neuroblastoma cell line SH-SY5Y was purchased from American Type Culture Collection (USA). SH-SY5Y cells were cultured and passaged in Dulbecco's Modified Eagle Medium (DMEM) contain-ing with 10% fetal bovine serum (Gibco, Grand Island, NY, USA), 100 U/ml penicillin and 100 mg/mL streptomycin. MPP+ (500 uM) was used to treat SH-SY5Y cells for 24 h.

### Cell transfection

The HOTAIR expressed was up-regulated or down-regulated by the transfection of pcDNA-HOTAIR (pcDNA as control) or si-HOTAIR (si-NC) according to the manufacturer's instructions. Cells were transfected with pcDNA-HOTAIR or si-HOTAIR or corresponding controls using Lipofectamine 2000 (Invitrogen, USA) according to the instructions provided by the manufacturer. At 48 h post transfection, cells were harvested for qRT-PCR.

### RNA extraction and qRT-PCR

Total RNA was extracted from brain tissues or SH-SY5Y cells using Trizol reagent (Invitrogen, USA) according to manufacturer's instructions. Complementary DNA (cDNA) was synthe-sized with Reverse Transcription M-MLV (TaKaRa Biotechnology, Dalian, China). Primers used for HOTAIR amplification were: forward 5′-CAGTGGGGAACTCTGACTCG-3′ and reverse 5′-GTGCCTGGTGCTCTCTTACC-3′. The relative expression levels of HOTAIR *were* quantified by Applied Biosystems 7500 Real-Time PCR System (Applied Biosystems, USA) with Power SYBR1 Green PCR Master Mix (Applied Biosystems, USA) according to the supplier's protocol. The relative mRNA expression levels were analyzed and expressed relative to threshold cycle values (ΔCt), then converted to fold changes using the 2^−ΔΔCt^ method. GAPDH was used as an internal control.

### Western blot

The level of LRRK2 protein expression was evaluated using western blot. After harvesting the total protein from midbrain or cultured cells, the concentration of protein was detected by Bradford Protein Assay Kit (Beyotime, Shanghai, China). An equivalent protein in each sample was separated on the 10% sodium dodecyl sulfate polyacrylamide gel (SDS-PAGE) electro-phoresis, and electrotransferred to polyvinylidene fluoride (PVDF) membranes. After blocking with 5% milk in PBS-0.05% Tween, membranes were incubated with primary antibody against LRRK2 (1:1000, Cell Signaling Technology, Danvers, MA, USA) or β-actin (1:1000, Cell Signaling Technology, Danvers, MA, USA) overnight at 4°C. Followed by incubation with horseradish peroxidase-conjugated secondary antibodies for 1 h, the blots were visualized with a PowerOpti-ECL kit according to the recommended procedure and protein bands were quantified using NIH ImageJ software.

### MTT assay

The cell viability of SH-SY5Y cells was evaluated by the MTT assay. Cells were plated in a 96-well plate at 5 × 10^3^ cells/well and were allowed to grow for different times. The growth rate was determined by the cell number and was counted in triplicate every day by MTT assay. Briefly, cells were incubated with 50 μL of 0.2% MTT for 4 h at 37°C in a 5% CO_2_ incubator. Following MTT incubation, 150 μL of 100% DMSO was added to dissolve the crystals. Viable cells were counted every day by reading the absorbance at 490 nm using a 96-plate reader BP800 (Dynex Technologies).

### Apoptosis detection by flow cytometry

Apoptosis was determined using Annexin Vfluorescein isothiocyanate (FITC) and propridium iodide (PI) staining. Forty-eight hours after transfecting RNA oligonucleotides to the SHSY5Y cells with or without MPP+ treatment, the cells were harvested, centrifuged, and resuspended in binding buffer. Approximately 10 μL of ready-to-use Annexin V-FITC (BD Bioscience, MA, USA) was added into the mixture, incubated at 37°C for 15 min, and then counterstained with 5 μL PI in the dark for 30 min. Annexin V-FITC and PI fluorescence were assessed using BD FACSCalibur flow cytometer (BD Bioscience), and the results were analyzed using the CellQuest software (BD Bioscience).

### Quantitative caspase 3 activity assay

Caspase 3 activity assay was conducted using the Caspase 3/CPP32 Colorimetric Assay Kit (Biovision, Palo Alto, CA) following the standard protocols. Forty-eight hours after the SH-SY5Y cells with or without MPP+ treatment were transfected with siRNA, the cells were harvested, washed with cold PBS, and then incubated with 50 μL of chilled lysis buffer on ice for 10 min. After centrifugation at 10,000 × *g*, protein (150 μg) was added into 2 × 50 μL of reaction buffer containing 5 μL of *N*-acetyl-Asp-Glu-Val-AsppNA substrate (200 μM final concentration). After incubation for 1–2 h at room temperature, *N*-acetyl-Asp-Glu-Val-Asp-pNA cleavage was monitored using a microplate reader (Bio-Tek Instruments Inc., Winooski, VT, USA). The absorbance (405 nm) of each well was detected to monitor the enzyme-catalyzed pNA release.

### Statistical analysis

All data are expressed as the mean ± standard deviation. Multiple comparisons analyses were performed using a one-way analysis of variance followed by Tukey's *post-hoc* tests using the Statistical Package for the Social Sciences (SPSS) software version 16.0 (SPSS, Inc., Chicago, IL, USA). *P* < 0.05 was considered to indicate a statistically significant difference.
